# Molecular detection of *Leishmania* DNA and identification of blood meals in wild caught phlebotomine sand flies (Diptera: Psychodidae) from southern Portugal

**DOI:** 10.1186/s13071-015-0787-4

**Published:** 2015-03-23

**Authors:** Carla Maia, Ricardo Parreira, José Manuel Cristóvão, Ferdinando Bernardino Freitas, Maria Odete Afonso, Lenea Campino

**Affiliations:** Unidade de Parasitologia Médica, Global Health and Tropical Medicine (GHTM), Instituto de Higiene e Medicina Tropical (IHMT), Universidade Nova de Lisboa (UNL), Lisbon, Portugal; Unidade de Microbiologia Médica, GHTM, IHMT-UNL, Lisbon, Portugal; Departamento de Ciências Biomédicas e Medicina, Universidade do Algarve, Faro, Portugal

**Keywords:** Blood-meal, *Leishmania*, Phlebotomine sand flies, *Phlebotomus perniciosus*, *Sergentomyia minuta*, Portugal, Southwestern Europe

## Abstract

**Background:**

Zoonotic visceral leishmaniasis caused by *Leishmania infantum* which is transmitted by phlebotomine sand flies (Diptera, Psychodidae) is endemic in the Mediterranean basin. The main objectives of this study were to (i) detect *Leishmania* DNA and (ii) identify blood meal sources in wild caught female sand flies in the zoonotic leishmaniasis region of Algarve, Portugal/Southwestern Europe.

**Methods:**

Phlebotomine sand flies were collected using CDC miniature light traps and sticky papers. Sand flies were identified morphologically and tested for *Leishmania* sp. by PCR using ITS-1 as the target sequence. The source of blood meal of the engorged females was determined using the *cyt-b* sequence.

**Results:**

Out of the 4,971 (2,584 males and 2,387 females) collected sand flies, *Leishmania* DNA was detected by PCR in three females (0.13%), specifically in two specimens identified on the basis of morphological features as *Sergentomyia minuta* and one as *Phlebotomus perniciosus*. Haematic preferences, as defined by the analysis of *cyt-b* DNA amplified from the blood-meals detected in the engorged female specimens, showed that *P. perniciosus* fed on a wide range of domestic animals while human and lizard DNA was detected in engorged *S. minuta*.

**Conclusions:**

The anthropophilic behavior of *S. minuta* together with the detection of *Leishmania* DNA highlights the need to determine the role played by this species in the transmission of *Leishmania* parasites to humans. In addition, on-going surveillance on *Leishmania* vectors is crucial as the increased migration and travelling flow elevate the risk of introduction and spread of infections by *Leishmania* species which are non-endemic.

## Background

Leishmaniasis caused by *Leishmania infantum* is the only tropical vector-borne disease that has been endemic in southern Europe for decades [1]. Most of the reported cases are due to zoonotic visceral leishmaniasis (VL), the most dangerous form of *Leishmania* infection, being lethal when untreated. Dogs are considered the major host for these parasites, and the main reservoir for human infections. In nature, the pathogen transmission occurs via the infective bite of phlebotomine sand flies (Diptera, Psychodidae), for both humans and dogs.

In Portugal, as in other countries in the south of Europe, VL was initially described as a pediatric disease but from the end of the 1980s onwards, the number of cases in children has decreased with a concomitant increase of infection in adults, commonly associated with HIV/AIDS [2]. In the last ten years (2005–2014), 119 new cases of human VL (17 in immunocompetent adults, 36 in children and 66 in immunocompromised patients) and 16 cutaneous leishmaniasis cases were diagnosed at the Leishmaniasis Laboratory at the Institute of Hygiene and Tropical Medicine. *Leishmania infantum* zymodeme MON-1 is the most common aetiological agent of autochthonous human and canine leishmaniasis cases [3] and *Phlebotomus perniciosus* and *Phlebotomus ariasi* have been confirmed as proven vectors [4].

As data regarding *Leishmania* infection rate and blood meal sources of phlebotomine sand flies in Portugal is still too scarce, this study was implemented so as to allow the (i) detection of *Leishmania* DNA and (ii) identification of blood meal sources in wild caught female sand flies in Algarve, Portugal/Southwestern Europe.

## Methods

### Study area

Algarve, located in southern Portugal, has an area of 5,412 Km2 with an estimated number of permanent inhabitants approximating 450,000 [5], which triplicates during summer months. Figs (*Ficus carica*), almonds (*Prunus amygdalus*), oranges (*Citrus sinensis*), carobs (*Ceratonia siliqua*), strawberries trees (*Arbutus unedo*) and cork oaks (*Quercus suber*), are the most common crops in the region [6]. Algarve has a Mediterranean climate with warm weather (annual average temperature of 18°C) and low rainfall almost all year round (annual average of 500 mm). Summer (June-September) is the driest and warmest season with average monthly temperatures between 16° and 28-30°C (www.ipma.pt).

### Collection and identification of sand flies

Between May to October from 2011 to 2013, CDC light traps and sticky oil papers were set up in 11 sampling points during three consecutive days per month. Collection places included domestic, peri-domestic and sylvatic environments. In most of the studied biotopes, in addition to humans and dogs, the major vertebrates visible within a 50 m radius of the collection spots were livestock, horses, pigs, rabbits and poultry. Collected sand flies were stored in 70% ethanol for further analysis. A total of 4,971 sand flies (2,584 males and 2,387 females) were collected and identified morphologically. Phlebotomine specimens of both genders were identified by their morphological characteristics to the species level, according to Pires [7]. Female identification was done by microscopic observation of the spermatheca, after dissection and mounting of the three last abdominal segments in Marc-André solution, while males were identified by direct stereomicroscopic observation of the genitalia. In addition, for each female, the presence of eggs (gravid status), and/or blood (engorged: total or partial vs. unfed) in the abdomen was recorded (Table 1).

**Table 1:** **Sand fly specimens collected according to the capture method and their positivity to** ***Leishmania*** **spp.**

	**CDC light traps**		**Sitcky papers**		**Total**			**Females**		
**Sand fly species**	**Females**	**Males**	**Females**	**Males**	**Females**	**Males**	**Females + Males**	**Blood fed**	**Gravid**	**Positive**
*Phlebotomus ariasi*	34	8	0	4	34	12	46	3	2	0
*Phlebotomus perniciosus*	372	305	59	806	431	1111	1542	49	32	1
*Phlebotomus papaptasi*	1				1		1		1	
*Phlebotomus sergenti*	27	26	27	94	54	120	174	1	1	0
*Sergentomyia minuta*	212	149	1655	1192	1867	1341	3208	25	49	2
**Total**	646	488	1741	2096	2387	2584	4971	78	85	3

### DNA extraction, PCR amplification and DNA sequencing

For each female sand fly, the remainder of the body (minus genitalia) was used as the source of DNA, extracted using the Citogene® Cell and Tissue kit (Citomed, Portugal) following the manufacturer’s instructions, with the exception that the maceration of the insect’s tissues was carried out with a piston pellet, and the final elution volume was 30 μl.

The PCR amplification of the internal transcribed spacer 1 (ITS-1) of the ribosomal operon of *Leishmania* was performed using the LITSR and L5.8S primers generating amplicons with 300–350 bp [8]. A positive control containing *L. infantum* DNA (MHOM/PT/88/IMT151) and a negative control without DNA template were included. To identify the origin of the blood meal of engorged females, the modified vertebrate-universal specific primers (cytB1-F and cytB2-R) were used to amplify a 350 bp segment of the host mitochondrial *cytochrome b* gene (*cyt-b*) [9]. PCR amplifications were performed in a 25 μl final volume containing 12.5 μl of NZYTaq 2× Green Master Mix (Nyztech, Portugal), 1 μl of each primer (10 pmol) and 2 μl of template DNA. The cycling profile used for the amplification of ITS-1 sequences included an initial denaturation step at 95°C for 2 min, followed by 32 repeats of 95°C-20 sec, 53°C-30 sec, 72°C-1 min followed by a final extension step at 72°C-6 min, while the preparation of *cyt-b* PCR products was carried out starting from 95°C for 5 min, followed by 40 cycles of 94°C-1 min, 55°C-1 min, 72°C-1 min followed by 72°C-7 min. Both amplicons were visualized under UV illumination after their resolution by conventional electrophoresis on 1.5% agarose gels stained with Greensafe premium® (Nzytech, Portugal), using a 100 bp DNA ladder as a molecular weight marker. PCR products were purified with a High Pure PCR Product Purification Kit (Roche® Mannheim, Germany) according to the manufacturer’s instructions. Subsequently, purified products were sent to LIGHTrun^TM^ Sequencing Service (GATC-biotech, Germany) for direct sequencing by Sanger’s method with the same primers used for DNA amplification.

### DNA sequence analyses

The identity of the feeding host (species level), carried out on the basis of the analysis of the obtained *cyt-b* sequences, was determined according to the closest BLASTn match (identity ≥ 99%) to a homologous sequence deposited at GenBank. The sequences obtained in the course of this work were deposited at DNA Data Bank of Japan (DDBJ) (http://www.DDBJ.nig.ac.jp).

Restriction profile was obtained by virtual digestion for ITS-1 sequence by using the Restriction Mapper (version 3 available online at http://www.restrictionmapper.org/).

Phylogenetic relationships were inferred from ITS-1 nucleotide sequence alignments produced with the MAFFT multiple alignment program using a combination of the Q-INS-i and E-INS-i alignment options [10]. Phylogenetic tree construction was carried out using a Maximum Likelihood (ML) approach, and the Kimura’s 2-P (K2P) evolutionary model, also assuming Γ distributed substitution rates among sites, as indicated by Mega6 [11] and as defined by the Akaike information criterion. Alternatively, an empirically defined model (GTR + Γ + I) was also used. The topological robustness of the obtained trees was assessed by bootstrapping, using 1000 resampling of the original alignment data. The final trees were manipulated for display using FigTree v.1.2.2. (available at http://tree.bio.ed.ac.uk/software/figtree/). NeighborNet networks (NNn) were constructed using the same distance matrix using Splits Tree4 software [12]; software available at http://www.splitstree.org/). Mean genetic distance values were calculated with the K2P formula, using Mega6 [11].

## Results

### Morphological identification of sand flies

*S. minuta* was the most prevalent species totaling a number of 3,208 specimens (64.53%), followed by *P. perniciosus* with 1,542 specimens (31.02%). *Phlebotomus sergenti* (174; 3.50%), *P. ariasi* (46; 0.93%) and one *P. papatasi* female (0.02%) were also collected. Eighty five females (2 *P. ariasi*, 1 *P. papatasi*, 32 *P. perniciosus*, 1 *P. sergenti* and 49 *S. minuta*) were gravid.

### *Leishmania* DNA detection, sequencing, and phylogenetic inference analysis

*Leishmania* DNA was detected in three apparently unfed females (0.13%) identified as *P. perniciosus* (n = 1) and in *S. minuta* (n = 2). The three positive females were collected in peridomestic biotopes (i.e. *P. perniciosus* was collected in a horse stable, and *S. minuta* were collected in a cattle pen and close to a kennel, respectively). The three ITS-1 obtained sequences were submitted to DDBJ (DDBJ accession numbers: LC028233 to LC028235). PCR product obtained from *P. perniciosus* had a similar size as *L. infantum* control while the PCRs products from both *S. minuta* were slightly bigger (data not shown). Furthermore, a *Hae*III restriction profile characteristic of *L. infantum* (184 bp, 72 bp and 55 bp) was obtained after virtual digestion of the ITS-1 sequence obtained from the positive DNA control as well as from *P. perniciosus*. Finally, sequence homology searches using BLASTn (megablast search option) revealed >99% identity with *L. infantum*, *L. chagasi* or *L. donovani* (E-values = e^−154^), and a sequence coverage >94%. Curiously, however, species assignment to the ITS-1 sequences amplified from *S. minuta* could not be carried out on the basis of nucleotide sequence homology search results. In this case the 15 best matches obtained with BLASTn (megablast) revealed >93% sequence identity (>95% sequence coverage and E-values < e^−122^) with only *Leishmania* sequences of Chinese origin referred to as *Leishmania* sp. [13], indicating relatively low identity with any sequence references already deposited in the sequence databases. Virtual *Hae*III restriction profiles of the ITS-1 sequences amplified from *S. minuta* (strains 5277 and 3400) were characterized by three DNA fragments (<193 bp, 89 bp, <54 bp), which were found to be similar, though not identical, to the virtual *Hae*III profiles determined for the Chinese *Leishmania* sp. sequences (<210 bp, 87 bp, <43 bp) mentioned above.

**Figure 1:**
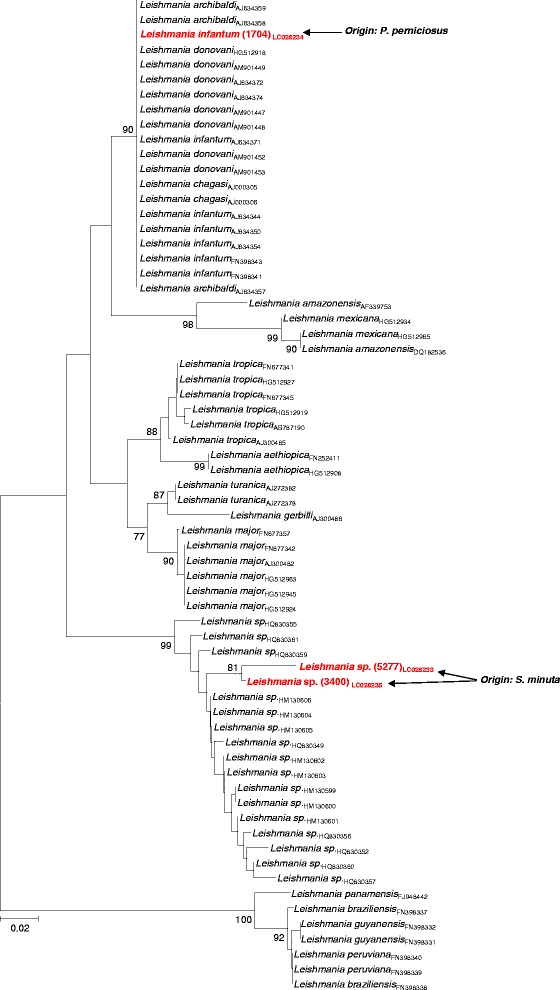
**Maximum likelihood phylogenetic tree (midpoint rooted) of** ***Leishmania*** **ITS-1 sequences amplified from phlebotomine sand flies collected in Portugal.** The percentages of significant (≥77%) bootstrap values of 1000 resamplings of the original data are indicated at specific branch-nodes. The size bar indicates 0.02 substitutions per site.

Definition of the species status of the obtained ITS-1 sequences was further pursued on the basis of phylogenetic analyses, along with others directly downloaded from the public database, and used as references (Table 2). The use of the suggested evolutionary model (K2P + Γ) or a more robust one (GTR + Γ + I), empirically defined by the user, resorted in phylogenetic trees with identical topologies as that shown in Figure 1 (data not shown).

**Table 2:** **Nucleotide reference sequences used in this work**

Species*	**Strain/** **isolate/** **haplotype**	**Origin/** **host**	**Accession number**
*Leishmania donovani*	MHOM/KE/83/NLB189	Kenya/Human	AJ634374
*Leishmania donovani*	MHOM/SD/93/9S	Sudan/Human	AJ634372
*Leishmania donovani*	MHOM/LK/2002/L60c	Sri Lanka/Human	AM901447
*Leishmania donovani*	MHOM/LK/2002/L60b	Sri Lanka/Human	AM901448
*Leishmania archibaldi*	MHOM/SD/93/GE	Sudan/Human	AJ634357
*Leishmania archibaldi*	MHOM/SD/97/LEM3429	Sudan/Human	AJ634358
*Leishmania archibaldi*	MHOM/SD/97/LEM3463	Sudan/Human	AJ634359
*Leishmania donovani*	MHOM/SU/84/LEM0946	Soviet Union/Human	HG512918
*Leishmania donovani*	MCAN/MA/2002/AD3	Morocco/Canine	AM901453
*Leishmania donovani*	MHOM/IQ/1981/SUKKAR2	Iraq/Human	AM901452
*Leishmania donovani*	MHOM/IN/1983/CHANDIGARH	India/Human	AM901449
*Leishmania infantum*	MCAN/UZ/2007/LRC-L1309	Uzbekistan/Canine	FN398341
*Leishmania infantum*	MHOM/BR/2007/JFF BM	Brazil/Human	FN398343
*Leishmania infantum*	MHOM/IT/93/ISS800	Italy/Human	AJ634354
*Leishmania infantum*	MHOM/PT/00/IMT260	Portugal/Human	AJ634344
*Leishmania infantum*	MHOM/MT/85/BUCK	Malta/Human	AJ634350
*Leishmania infantum*	MHOM/SD/93/452BM	Sudan/Human	AJ634371
*Leishmania chagasi*	MHOM/BR/85/M9702	Brazil/Human	AJ000306
*Leishmania chagasi*	MHOM/PA/79/WR317	Panama/Human	AJ000305
*Leishmania tropica*	MHOM/IL/01/LRC-L838	Israel/Human	FN677341
*Leishmania tropica*	MHOM/EG/90/LPN65	Egypt/Human	HG512927
*Leishmania tropica*	MHOM/PS/01/ISL590	Palestine*/Human	FN677345
*Leishmania tropica*	MHOM/YE/86/LEM1015	Yemen/Human	HG512919
*Leishmania tropica*	MHOM/TN/88/TAT3	Tunisia/Human	AJ300485
*Leishmania tropica*	IHAM/GH/2007/KLE-18	Ghana/*Sergentomyia hamoni*	AB787190
*Leishmania aethiopica*	MHOM/ER/2009/7457	Eritrea/Human	FN252411
*Leishmania aethiopica*	MHOM/KE/71/KPS-H2	Kenya/Human	HG512908
*Leishmania turanica*	KD85001	Uzbekistan/*Rhombomys opimus*	AJ272378
*Leishmania turanica*	KL3	Kazakhstan/*Rhombomys opimus*	AJ272382
*Leishmania gerbilli*	MRHO/UZ/87/KD-87555	Uzbekistan/*Rhombomys opimus*	AJ300486
*Leishmania major*	MTAT/KE//NLB089A	Kenya/ND	AJ300482
*Leishmania major*	MHOM/UZ/02/17h	Uzbekistan/Human	FN677357
*Leishmania major*	MHOM/BF/2004/REN04-8	Burkina Faso/Human	HG512963
*Leishmania major*	MHOM/JO/90/JH39	Jordan/Human	HG512945
*Leishmania major*	MHOM/TN/97/LPN162	Tunisia/Human	FN677342
*Leishmania major*	MHOM/DZ/89/LIPA228	Algeria/Human	HG512924
*Leishmania mexicana*	MHOM/PE/02/LH2312	Peru/Human	HG512965
*Leishmania mexicana*	MHOM/EC/90/LM	Ecuador/Human	HG512934
*Leishmania amazonensis*	MHOM/BR/73/M2269	Brazil/Human	DQ182536
*Leishmania amazonensis*	IFLA/BR/67/PH8	Brazil/ND	AF339753
*Leishmania braziliensis*	MHOM/PE/2003/LH2920	Peru/Human	FN398337
*Leishmania braziliensis*	MHOM/BR/00/LTB300	Brazil/Human	FN398338
*Leishmania peruviana*	MHOM/PE/2006/LH3667	Peru/Human	FN398340
*Leishmania peruviana*	MHOM/PE/1990/HB86	Peru/Human	FN398339
*Leishmania guyanensis*	MHOM/BR/2002/NMT-RBO013	Brazil/Human	FN398331
*Leishmania guyanensis*	MHOM/PE/2006/LH3635	Peru/Human	FN398332
*Leishmania panamensis*	Isolate 18, clone 4	ND/Human	FJ948442
*Leishmania* sp*.*	MHOM/CN/80/XJ801	P.R.China/Human	HQ830357
*Leishmania* sp*.*	MHOM/CN/89/GS5	P.R.China/Human	HQ830360
*Leishmania* sp*.*	MHOM/CN/90/SC10H2	P.R.China/Human	HQ830352
*Leishmania* sp*.*	MHOM/CN/86/SC6	P.R.China/Human	HQ830356
*Leishmania* sp*.*	MHOM/CN/90/SC10H2	P.R.China/Human	HM130601
*Leishmania* sp*.*	MCAN/CN/60/GS1	P.R.China/Canine	HM130600
*Leishmania* sp*.*	MHOM/GS6/CHN/SCgq	P.R.China/Human	HM130599
*Leishmania* sp*.*	MCAN/CN/86/SC9	P.R.China/Canine	HQ830359
*Leishmania* sp*.*	MHOM/CN/83/GS2	P.R.China/Human	HM130603
*Leishmania* sp*.*	MHOM/GS5/CHN/SCH2g	P.R.China/Human	HM130602
*Leishmania* sp*.*	MHOM/SC11/CHN/SCgz	P.R.China/Human	HM130606
*Leishmania* sp*.*	MHOM/CN/84/JS1	P.R.China/Human	HM130605
*Leishmania* sp*.*	MHOM/CN/84/SD1	P.R.China/Human	HM130604
*Leishmania* sp*.*	MHOM/CN/89/GS6	P.R.China/Human	HQ830355
*Leishmania* sp*.*	MHOM/CN/90/SC11	P.R.China/Human	HQ830361

*Species as defined by the depositors; Israel: Occupied Palestinian Territories; P. R. China: People’s Republic of China; ND: not defined.

**Figure 2:**
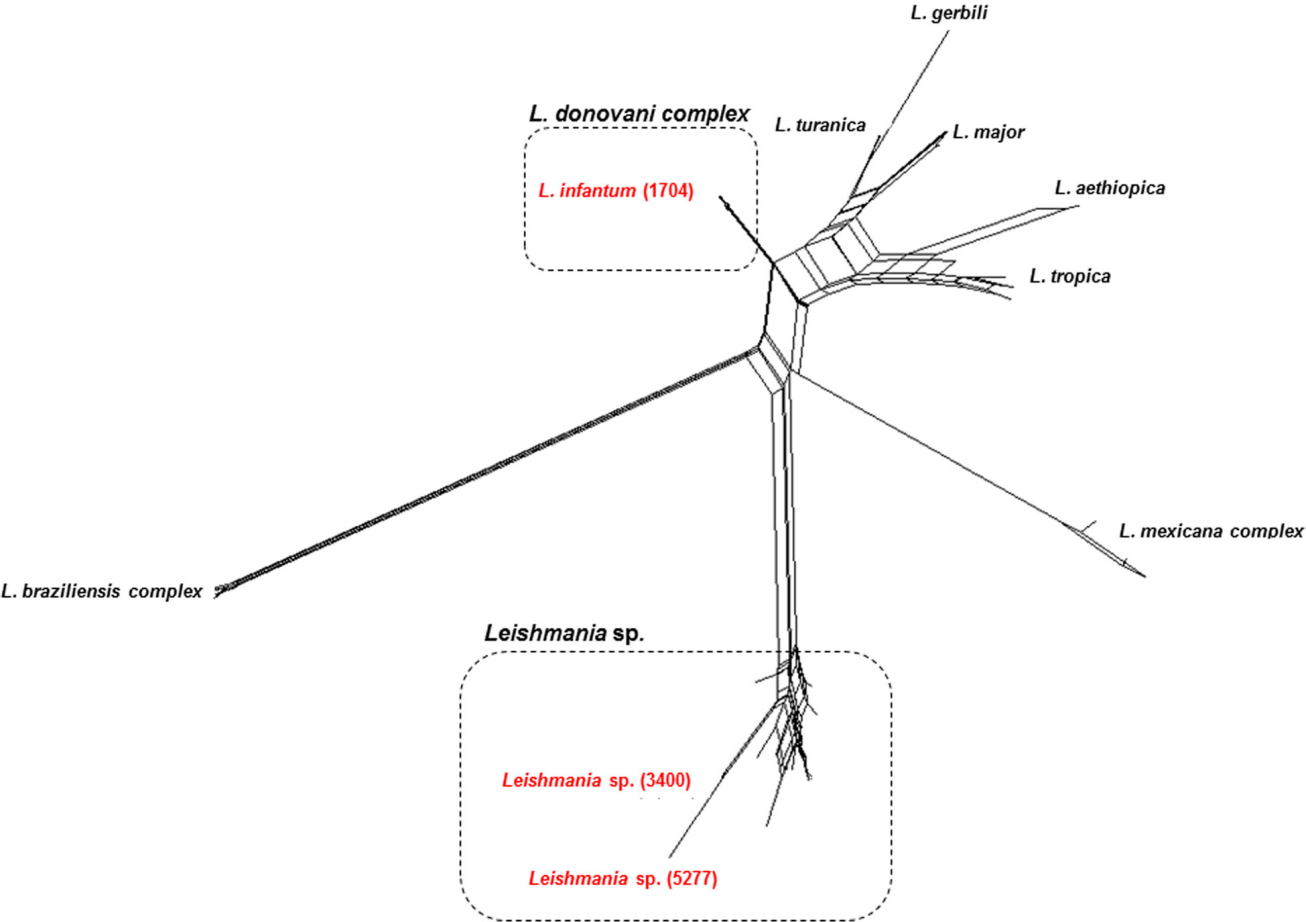
**NeighborNet network constructed with SplitsTree software employing the matrix of genetic distances (corrected with the K2P formula) between individual** ***Leishmania*** **ITS-1 sequences amplified from phlebotomine sand flies collected in Portugal, and reference sequences.**

One of the sequences obtained in this study (strain 1704), amplified from *P. perniciosus*, was found to segregate in a large monophyletic cluster that included *L. infantum*, *L. donovani, L. archibaldi* and *L. chagasi* (Figure 1), characterized by low genetic variability (average genetic distance of 0.2%). On the other hand, the remainder two ITS-1 sequences (strains 5277 and 3400), amplified from *S. minuta*, were found to locate in a bootstrap-supported (99%) assemblage of multiple reference sequences of Chinese origin, merely defined as *Leishmania* sp. [13], and that included a multitude of *Leishmania* sequences from human and canine origin, with an average genetic distance of 2.6% (ranging from 0% to 8.0%), indicating considerably higher genetic variability than that associated with the *L. infantum/L. donovani/L. archibaldi/L. chagasi* cluster. Similar conclusions were achieved when, instead of assuming a strict tree-like evolution, the phylogenetic relationships between ITS-1 sequences were represented as a NNn (Figure 2).

### Vertebrate DNA detection in female sand flies

A total of 78 engorged female sand flies (3 *P. ariasi*, 49 *P. perniciosus*, 1 *P. sergenti* and 25 *S. minuta*) were tested to determine the vertebrate host source of the blood meal. A positive PCR amplification result was obtained for 43 of the collected specimens. After DNA sequencing of the amplified partial *cyt-b* sequences, the origin of 30 (69.77%) blood-meals was identified (Table 3) on the basis of the closest sequence matches, as defined by BLASTn sequence homology searches (>99% identity with deposited at the GenBank/EMBL/DDBJ public databases).

**Table 3:** **Identification of sand fly blood meal sources**

**Sand fly host**	***P*** *.* ***ariasi***	***P. perniciosus***	***S. minuta***	**Blast identity for the blood meal**	**DDBJ accession no.**						
Horse *(Equus caballus)*	0	12	0	99-100%	AB985687	AB985693-97	AB985699	AB985703	AB985708	AB985711	AB985714
Chicken *(Gallus gallus)*	0	5	0	99-100%			AB985704	AB985705	AB985710	AB985713	AB985715
Human *(Homo sapiens)*	0	0	4	99-100%				AB985688	AB985689	AB985698	AB985712
Rabbit (*Oryctolagus cuniculus*)	0	3	0	99%					AB985690	AB985700	AB985709
Pig (*Sus scrofa*)	0	2	0	99						AB985707	AB985716
Cattle (*Bos taurus*)	1	1	0	99						AB985702	AB985706
Sheep (*Ovis aries*)	0	1	0	99							AB985701
Lizard (*Tarentola mauritanica*)	0	0	1	99							AB985692
**Total**	1	24	5								

## Discussion

Phlebotomine sand flies are distributed in all countries around the Mediterranean basin, turning both human populations and domestic animals living in these areas into potential targets to sand fly-borne diseases such as leishmaniasis. Therefore, knowledge on the host preferences of sand flies under natural conditions is essential not only to understand their vectorial role, but also as a means to identify potential reservoir hosts. In this work, we detected *Leishmania* DNA and evaluated blood meal sources of fed females sand flies captured in southern Portugal, where zoonotic leishmaniasis is known to be endemic [2].

Similarly to what has been observed by others [14-19] the blood meal analysis of the engorged *P. perniciosus* revealed that this species fed on a broad variety of vertebrates hosts (i.e. horses, cattle, sheep, pigs, rabbits and chickens) highlighting its opportunistic feeding behaviour. Interestingly, no dog or human blood was detected in blood-fed *P. perniciosus*, despite the fact that it has been clearly defined as a proven vector of *L. infantum* in the Algarve region [19-21]. The apparent absence of *P. perniciosus* feeding on dogs and humans might indicate that in the sampled biotopes, neither of them were the main blood sources for this sand fly species due to the presence of other larger sized vertebrates (e.g. horses) and/or present in greater numbers (i.e. chicken, rabbits), making them easier targets.

In addition, *Leishmania infantum* DNA was detected in one unfed *P. perniciosus* specimen. Assignment of species status for the 1704 ITS-1 sequence could not be clearly carried out solely based on phylogenetic tree analysis due to the low genetic variability of the ITS-1 sequences that define the *L. donovani* complex [22] (that include *L. infantum, L. donovani, L. archibaldi* and *L. chagasi*, precluding a clear resolution of this genetic cluster, as previously observed [13]. Nevertheless, the ITS-1 amplicon size and virtual *Hae*III restriction profile obtained for the 1704 sequence amplified from *P. perniciosus* were compatible with it corresponding to *L. infantum*, and reinforces the maintenance of this sand fly species as vector of *L. infantum* in southern Portugal [19-21].

Sand flies of the *Sergentomyia* genus, which is widely distributed throughout the Old World, are proven vectors of reptile *Leishmania* species [23]. It is generally accepted that most of *Sergentomyia* species are not anthropophilic, and as a consequence cannot transmit either *Leishmania* or any other pathogens to humans. However, in the present study, apart from detecting *Tarentola mauritanica* (a reptile widely distributed around the Mediterranean area [23,24]) DNA in one engorged *S. minuta*, human DNA was also amplified in four specimens corroborating that at least some *Sergentomyia* species disclose sporadic/opportunistic anthropophilic feeding-behaviour [25,26]. Furthermore, *Leishmania* sp. DNA was detected in two unfed *S. minuta* females, which unambiguously allocated with references within a cluster of Chinese *Leishmania* sp. previously isolated from humans and canine leishmaniasis cases [13]. While phylogenetic tree reconstruction and NNn analyses showed that the two ITS-1 sequences amplified from *S. minuta* (strains 5277 and 3400) clearly segregated away from all the others in a genetically consistent assemblage of *Leishmania* strains, in this case species assignment was limited by the unavailability of well characterized reference strains. However, despite the inability to clearly define the species of origin of the obtained sequences using phylogenetic analyses, the detection of *Leishmania* DNA phylogenetically related to those considered pathogenic to humans and dogs in China [13] was somewhat unexpected.

According to Yang et al. [13], the above mentioned *Leishmania* strains of Chinese origin belonged to an undefined species, that was found to be genetic divergent from any of the known New and Old World *Leishmania*, on the basis of ITS-1 sequence analysis. Similar results were obtained when kinetoplast cytochrome oxidase II (COII; [27]) or CYT-b coding sequences [28] amplified from these same strains were analysed. Interestingly, both phylogenetic inference reconstruction studies revealed that the Chinese *Leishmania* sp. isolates were most closely related to the lizard-infecting *L. tarentolae*. Unfortunately, in the present study it was not possible to evaluate if the two *Leishmania* sp. detected in *S. minuta* were genetic related to this reptile *Leishmania* species, as no ITS-1 sequences of *L. tarentolae* have yet been deposited in DNA sequence databases for public access. On the other hand, exhaustion of the DNA extracts on which the analysis presented in this report was based ruled out any possibility of generating *cyt-b* and/or *coII* sequence data. Nevertheless, and taking into account the results obtained with *cyt-b/coII* [27,28], in the near future it will be important to analyse more of these *Leishmania* parasites obtained from both vertebrate (including reptiles) and invertebrate infected hosts for assessment of the parasite species as well as to determine their clinical significance, and estimate the potential risk their endemic establishment in Portugal/Europe. Ideally, should the laboratory settings allow it, further genetic analysis-based studies should be supported, as much as possible, by sequence datasets combining information from multiple genetic *loci*, so as to tentatively increase the phylogenetic signal, and achieve a better resolution of the observed genetic clusters, including the *L. donovani* complex [29].

Based upon literature reviews, a consideration of the role of *Sergentomyia* in the circulation of mammalian leishmaniasis becomes apparent as *Leishmania* DNA has been identified in several species. These include the molecular detection of *L. major* in *S. sintoni* in Iran [30], *S. garnhami* in Kenya [31], *S. darlingi* in Mali [25], and *S. minuta* in Portugal [32]. Furthermore *L. donovani* has been detected in *S. babu* in India [33], *L. infantum* in *S. dubia*, *S. magna* and *S. schewtzi* in Senegal [34], and *L. siamennsis* in *S. gemmea* in Thailand [35]. Finally, more recently, *L. tropica* has been found in *S. ingrami* and *S. hamoni* in Ghana [26]. Nevertheless, PCR positivity alone should not be used for incrimination of *Sergentomyia* sand flies as *Leishmania* vectors since the detection of DNA does not give any information about the parasites’ viability or its presence as virulent metacyclic promastigotes [36,37]. In fact, and although *L. infantum* DNA had been detected in *S. schwetzi* from Senegal [34], the refractoriness of this African species to some *Leishmania* species infecting humans (including *L. donovani, L. infantum* and *L. major*) has also been recently demonstrated [38]. In any case, the refractoriness of this particular *Sergentomyia* species does not necessarily extend to the whole of the genus. In this line of reasoning, the competence and permissiveness of the different species from *Phlebotomus* spp. to different Old World *Leishmania* has also been observed [39]. As *L. major* DNA had previously been detected in one *S. minuta* captured in the same region [32], together with the detection in this study of both human and *Leishmania* sp. DNA in this species, it would be important to determine if *S. minuta* fulfils the criteria that support its incrimination as vector for this parasite, and that include (i) the isolation of metacyclic promastigotes from the digestive tubes of field-collected specimens; and (ii) the experimental demonstration of its capacity to transmit Old World *Leishmania* species with medical and veterinarian importance as a result of blood-feeding on mammals.

## Conclusion

The apparent anthropophilic behavior of *S. minuta* together with the detection of *Leishmania* sp. DNA highlight the need to determine the role played by this sand fly species in the transmission of pathogenic *Leishmania* to humans. In addition, our data confirms that *P. perniciosus* is an opportunistic feeder and suggest that is responsible for the maintenance of *L. infantum* in sourthern Portugal. Altogether, the obtained results reinforce the need for on-going surveillance with systematic epidemiologic surveys on *Leishmania* vectors so as to investigate the transmission, distribution and spread of infections by *Leishmania* species.
